# Ultrasound Doppler fetal heart rate detection algorithm analyzes the correlation between twin selective fetal growth restriction and cord blood SFass fasL level

**DOI:** 10.12669/pjms.37.6-WIT.4881

**Published:** 2021

**Authors:** Qiaohong Cao, Cong Ma, Junbiao Zhu

**Affiliations:** 1 Qiaohong Cao, Bachelor’s Degrees. Department of Obstetrics and Gynecology, Wenling Maternal and Child Health Care Hospital, Wenling, 317500, Zhejiang, China; 2 Cong Ma, Bachelor’s Degrees. Department of Obstetrics and Gynecology, Wenling Maternal and Child Health Care Hospital, Wenling, 317500, Zhejiang, China; 3 Junbiao Zhu, Bachelor’s Degrees. Department of Obstetrics and Gynecology, Wenling Maternal and Child Health Care Hospital, Wenling, 317500, Zhejiang, China

**Keywords:** Cord Blood SFass FasL Level, Twin Selective Fetal Growth Restriction, Ultrasound Doppler

## Abstract

**Objective:**

The paper uses ultrasound Doppler fetal heart rate detection algorithm to explore the placental characteristics of monochorionic twin pregnancy with selective fetal growth restriction, and discuss the correlation between selective fetal growth restriction and cord blood SFass FasL level.

**Methods:**

From June 1, 2019 to June 1, 2020 in our hospital, 23 cases of selective fetal growth restriction and 32 cases of uncomplicated cases were included in the monochorionic twin pregnancies whose pregnancy was terminated in our hospital (control group) research. Perfusion was completed within 24 hours after delivery of the placenta. The umbilical arteries and veins of the two fetuses were respectively perfused with four different colors of pigments. The type of anastomoses was judged according to the color of the blood vessels on the placenta surface.

**Results:**

The selective fetal growth restriction group was higher than the control group. In the selective fetal growth restriction group and the control group, the number of anastomoses of the placental superficial arterial artery, arterial vein and venous vein were 1.0 and 1.0, 3.0 and 2.0, 0.0 and 0.0, respectively; the placental superficial arterial artery, arterial vein and venous vein. The total diameters of the anastomosed blood vessels were 2.7 and 2.2, 4.0 and 3.4, 0.0 and 0.0 mm, respectively; the total number of superficial placental anastomosed blood vessels in the selective fetal growth restriction group and the control group were 3.5 and 3.5, respectively.The total diameters were 6.9 and 6.9, respectively 5.9mm.

**Conclusion:**

Uneven placental share and non-central attachment of the umbilical cord may be risk factors for selective fetal growth restriction in monochorionic twin pregnancy.

## INTRODUCTION

Selective fetal growth restriction (sFGR) is one of the complications of monochorionic twin pregnancy, and its incidence is 10%~15%. The incidence of sFGR is related to factors such as the share of twin placenta and the location of umbilical cord. At present, foreign countries implement intrauterine interventions on placental structure for sFGR, but in China still mainly adopts expectant treatment or selective fetal reduction surgery, and there are few studies on sFGR placental structure. Superficial vascular perfusion was performed on the placenta of monochorionic twin pregnancy to explore the placental characteristics of sFGR.

## METHODS

From June 1st, 2019 to June 1st, 2020, the number of placentae, the number of amniotic sacs, and the number of septal layers were checked for twin pregnancies whose pregnancy was terminated in our hospital after delivery with IRB approval (dated March 23, 2021). A total of 97 cases were determined to be monochorionic. We excluded 18 cases of placental damage after delivery or fetal laser treatment during pregnancy, and 24 cases of twin-to-child transfusion syndrome. Twenty three cases of sFGR and 32 cases of uncomplicated patients (control group) were included for the study Their ages were between 23-45 years. sFGR refers to a single-chorionic twin pregnancy that is small for gestational age (SGA), that is, the ultrasound estimated fetal weight is below the 10th percentile of the corresponding gestational week, except for cases where both fetuses are SGA.^1^ SFGR is divided into three types: Type-I with normal diastolic blood flow; Type-II with continuous disappearance or reversal of diastolic blood flow; Type-III with intermittent end-diastolic disappearance or reversal of blood flow.

### Treatment of fresh placenta

The placenta is placed on a flat tray two hours after delivery, and the umbilical cords of the first and second fetuses are marked with straight forceps and curved forceps respectively. Wipe off blood stains on the surface of the placenta, and gently squeeze the blood vessels on the surface of the placenta along the blood vessels, squeeze most of the blood or fresh thrombus from the broken end of the umbilical cord. Peel off the fetal membrane on the surface of the chorionic plate and trim the umbilical cord to a length of 4 to 5 cm. Separate the broken ends of the blood vessel, and insert the trocar along the broken ends of the umbilical artery and vein respectively [2]. Only one umbilical artery needs to be cannulated. A 16G trocar is used for the umbilical vein; a 22G trocar is used for most of the umbilical arteries, and the umbilical cord is thin in some abortions, and a 26G trocar is used for the umbilical artery. The trocar was fixed with silk thread, and physiological saline was slowly injected to clean the remaining blood in the blood vessel. There was no blood or thrombus in all blood vessels on the surface of the placenta.The perfusion fluid is a 1:10 mixture of four watercolor paints: white, green, red, and black.

Complete perfusion within 24 hours of placental delivery. Lay the treated placenta flat on the Go board, and use a 20ml syringe to inject 15-20ml of perfusate along the cannula. The umbilical arteries and veins of the first fetus were infused with white and black pigments, and the umbilical arteries and veins of the second fetus were infused with red and green pigments. The artery is perfused first, and then the vein is perfused until the distal end of each blood vessel is filled. After the perfusion is completed, the umbilical cord is clamped with a vascular clamp. Take pictures perpendicular to the plane of the placenta.

Use professional graphics processing software Image Proclus Version 6.0 for Windows to determine the type of anastomosed blood vessel based on the color of the placental surface blood vessel. The arterial artery and venous anastomosis occurred on the surface of the chorionic plate. After perfusion, the two colors were confused and easy to distinguish. Arteriovenous anastomosis occurs in the chorionic lobules deep in the chorionic plate. What is seen on the surface of the chorionic plate is that a fetal artery without veins enters the chorionic plate, and another fetal vein without arteries enters the chorion. board.

### Analysis index:

(1) The placental area difference ratio.
(2) Location of umbilical cord attachment.
(3) The type, number and diameter of anastomoses.

### Ultrasound Doppler fetal heart rate detection algorithm

In the ultrasonic Doppler fetal heart rate monitoring system, the ultrasonic sensor transmits ultrasonic waves into the mother and fetus, encounters the moving fetal heart, and causes the echo signal to produce a frequency shift. The size of the frequency offset is related to the movement speed of the reflection interface, and the frequency offset *f_D_* is:


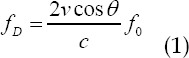


**Fig.1:**
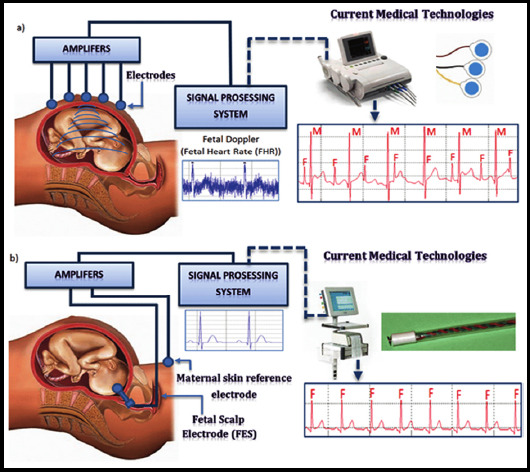
Block diagram of the fetal heart rate monitoring system.

Fig.1 shows the overall block diagram of the fetal heart rate monitoring system based on TMs32oe54oZDsp. The DSp is used to complete the heart rate sampling autocorrelation calculation. DSP transmits the data of fetal heart rate to AT89C52 single-chip microcomputer through serial communication, and AT89c52 outputs these data in an appropriate manner according to requirements, such as displaying or printing out on LcD. The peripheral interface circuit of AT89C52 is realized by MAX7128 programmable logic device.

Since the echo signal is a random signal, the detection, identification and extraction of the random signal can be realized through the method of correlation function. When the total number of data is N, the formula for autocorrelation estimation is:


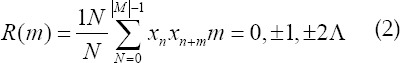


When calculating using formula (2), in order to achieve real-time detection, generally adopt methods such as reduced sampling frequency or autocorrelation approximate calculation such as absolute difference method. Taking into account the particularity of autocorrelation applied to fetal heart rate detection, in the process of calculating the fetal heart rate value through the maximum value of the autocorrelation for many times, we found that under a certain sampling frequency, a specific heart rate value is associated with a specific autocorrelation value. Corresponding serial numbers of the relevant results.

The calculation formula of the heart rate sampling coefficient is obvious, and the relationship between the heart rate coordinate value and the serial number of the autocorrelation result is shown in the following formula:


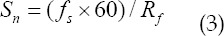


Among them, *S_n_* is the corresponding serial number, *R_f_* is the heart rate value, and *f_s_* is the sampling frequency. With this formula, the correspondence table between the heart rate value and the autocorrelation result serial number can be calculated. The specific calculation only needs to calculate the corresponding point by looking up the table. It can also be understood that the autocorrelation results are sampled with the serial number value in the table, and the autocorrelation results corresponding to the specific heart rate value are extracted, and the maximum value is found from it to determine the heart rate value.^3^,^4^

## RESULTS

There was no statistically significant difference between the sFGR group and the control group in the proportions of advanced-age mothers, trans-parturient, pregnancy through assisted reproductive technology, early pregnancy reduction, hypertension, diabetes mellitus in pregnancy, and gestational week of delivery (all P>0.05), Table-I. The gestational age of 23 cases in the sFGR group was> 28 weeks. In the control group, three cases were aborted in the second trimester due to premature rupture of membranes. In the sFGR group, the average gestational week onset was (26.6±4.2) weeks, with 14 cases of Type-I, 8 cases of Type-II, and one case of Type-III.

**Table-I:** Comparison of general conditions of 2 groups.

*Group*	*Cases*	*Senile parturient*	*Maternal*	*Pregnancy via assisted reproductive technology*	*Fetal reduction in early pregnancy*	*Hypertension*
SFGR	23	1(4.3)	2(8.7)	2(8.7)	0(0.0)	3(13.0)
Control	32	3(9.4)	7(21.9)	8(25.0)	1(3.0)	3(9.4)
P		0.632	0.277	0.166	1	0.686

### Placental area difference ratio

The birth weight of large fetuses in the sFGR group was higher than that of small fetuses (2119±454) and (1444±350), the difference was statistically significant (t=5.642, P=0.000). The birth weights of large fetuses and small fetuses in the control group were (2362±546) and (2112±553) g, respectively, and the difference was not statistically significant (t=1.567, P=0.091). Table-II.

**Table-II:** Comparison of placental area difference ratio and placental superficial anastomosis in the 2 groups.

*Group*	*Placental area ratio*	*Uneven placenta share*	*Total anastomoses*		*Arterial anastomosis*	

*Number*	*Total Diameter*	*number*	*Total Diameter*
SFGR	0.6	21	3.5	6.9	1.0	2.7
Control	0.2	16	3.5	5.9	1.0	2.2
Z	3.913	A	0.567	0.556	0.256	0.07
P	0	0.001	0.676	0.472	0.841	0.952
*Group*	*Arteriovenous anastomosis*			*Venous vein anastomosis*		

	*Example(%)*	*Number*	*Total Diameter*	*Example (%)*	*Number*	*Total diameter*
SFGR	19(82.6)	3.0	4.0	4	0.0	0.0
Control	23(71.9)	2.0	3.4	5	0.0	0.0

### Location and distance of umbilical cord attachment

The proportions of non-central umbilical cord attachment in sFGR for small fetuses, large fetuses and the control group were 82.6%, 13.0%, and 40.6%, respectively. The ratio of the distance between the two umbilical cord attachment points in the sFGR group and the control group to the longest diameter of the placenta was 0.55±0.26 and 0.59±0.21, respectively, and the difference was not statistically significant.

### Superficial vascular anastomosis of the placenta

The total number and total diameter of superficial placental anastomoses in the sFGR group, as well as the proportion, number and total diameter of arterial artery, arteriovenous and venous anastomoses, were not statistically different from those of the control group (all P>0.05). Table-II.

## DISCUSSION

The fetal heart rate refers to the number of times the fetus’s heart is regulated per minute, which is mainly used to assess the health status of the fetus during the perinatal period. Clinical studies have shown that the heart rate of a normal fetus ranges from 120 - 160 beats/min. When it exceeds this range, fetal distress occurs.^5^,^6^ At present, the most commonly used method for fetal heart rate detection is ultrasound Doppler.^7^ However, because the Doppler detected by the ultrasound Doppler fetal heart rate detector often contains a large amount of messy, large variability, and uneven noise, it will cause a large degree of interference to the calculation of fetal heart rate.^8^,^9^ The algorithms currently used for fetal heart rate detection by ultrasound Doppler are mainly divided into autocorrelation and short-term average amplitude difference method.^10^ The autocorrelation algorithm was adopted in this study to process the ultrasound Doppler of fetal heart rate, and the results showed that it can be used for the onset of fetal growth restriction during pregnancy, which provides a reference for understanding the degree of fetal growth restriction.

The fetus mainly supplies blood through the placenta to maintain its own growth and development.^11^ Abnormalities in the function and area of the placenta in singleton pregnancy directly cause the lack of nutrition and oxygen supply for fetal growth and development, and ultimately lead to fetal growth restriction.^12^ The diagnosis of fetal growth restriction in multiple pregnancies is relatively controversial. Based on the most commonly used clinical diagnosis of fetal growth restriction criteria, the follow-up research was developed in this study. At present, studies have shown that in twins with single chorionic and double amniotic sacs, the risk of fetal growth restriction caused by the imbalance of the placenta share is as high as 9.8 times.^13^ The difference in placenta area can also significantly affect the growth of the fetus.^14^ The results of this study showed that the proportion of placental difference and uneven share in the fetal growth restriction group was significantly higher than that of normal fetuses. In addition, when growth restriction occurs in twin fetuses, the birth weight of large fetuses was greatly higher than that of small fetuses. This may be due to the unevenness of the fetal placenta during pregnancy, which would result in insufficient fetal nutrition and oxygen supply, causing fetal growth restriction.^15^

## CONCLUSIONS

The results showed that the proportion, number and total diameter of the superficial arteries, arterial veins and venous veins of the placenta in the sFGR group, as well as the total number and total diameter of anastomoses, were not statistically different from the control group, suggesting anastomosis Blood vessels have no obvious influence on the pathogenesis of sFGR. Some researchers have shown that the anastomotic characteristics of the three types of sFGR may be different. The anastomotic vessels of Type-I sFGR are similar to those of uncomplicated monochorionic twins; only 18% of Type-I sFGR contain arterial anastomoses with a diameter> 2 mm. The small fetus has poor blood supply, slow weight gain, severe intrauterine hypoxia, and a high incidence of long-term brain injury; about 98% of Type-III sFGR have arterial anastomoses with a diameter of more than 2mm. In this study, there was only one case of Type-III sFGR, so the placental characteristics of the three types were not compared. Follow-up studies will expand the sample size and further explore the placental characteristics of different types of sFGR.

### Author`s Contribution:

**QC** conceived the study, literature review, data analysis, drafting the paper.

**CM** helped in design, data collection, article drafting & critical revision.

**JZ** takes the responsibility and is accountable for all aspects of the work in ensuring that questions related to the accuracy or integrity of any part of the work are appropriately investigated and resolved.

## References

[1] Khalil A, Beune I, Hecher K, Wynia K, Ganzevoort W (2019). Consensus definition and essential reporting parameters of selective fetal growth restriction in twin pregnancy:a Delphi procedure. Ultrasound Obst Gyn.

[2] Frusca T, Todros T, Lees C, Bilardo CM (2018). TRUFFLE Investigators Outcome in early-onset fetal growth restriction is best combining computerized fetal heart rate analysis with ductus venosus Doppler:insights from the Trial of Umbilical and Fetal Flow in Europe. Am J Obstet Gynecol.

[3] Fu B, Zhou Y, Ni X, Tong X, Xu X (2017). Natural Killer Cells Promote Fetal Development through the Secretion of Growth-Promoting Factors. Immunity.

[4] Warmerdam G, Vullings R, Van Laar J, der Jagt MBdHV, Bergmans JWM, Schmitt L (2018). Detection rate of fetal distress using contraction-dependent fetal heart rate variability analysis. Physiol Meas.

[5] Furuya N, Hasegawa J, Imai H, Homma C, Kurasaki A, Kondo H (2021). Accuracy of predicting neonatal distress using a five-level classification of fetal heart rate monitoring. J Obstet Gynaecol Res.

[6] Mdoe PF, Ersdal HL, Mduma ER, Perlman JM, Moshiro R, Wangwe PT (2018). Intermittent fetal heart rate monitoring using a fetoscope or hand held Doppler in rural Tanzania:a randomized controlled trial. BMC Pregnancy Childbirth.

[7] Hamelmann P, Vullings R, Kolen AF, Bergmans J, van Laar J, Tortoli P (2020). Doppler Ultrasound Technology for Fetal Heart Rate Monitoring:A Review. IEEE Trans Ultrason Ferroelectr Freq Control.

[8] Hayes-Gill BR, Martin T, Liu C, Cohen WR (2020). Relative accuracy of computerized intrapartum fetal heart rate pattern recognition by ultrasound and abdominal electrocardiogram detection. Acta Obstet Gynecol Scand.

[9] Kupka T, Matonia A, Jezewski M, Jezewski J, Horoba K, Wrobel J (2020). New Method for Beat-to-Beat Fetal Heart Rate Measurement Using Doppler Ultrasound Signal. Sensors (Basel).

[10] Yan Z, Xu X, Wang Y, Li T, Ma B, Yang L (2021). Application of Ultrasonic Doppler Technology Based on Wavelet Threshold Denoising Algorithm in Fetal Heart Rate and Central Nervous System Malformation Detection. World Neurosurg.

[11] Howel KR, Powell TL (2017). Effects of maternal obesity on placental function and fetal development. Reproduction.

[12] Burton GJ, Jauniaux E (2018). Pathophysiology of placental-derived fetal growth restriction. Am J Obstet Gynecol.

[13] Fick AL, Feldstein VA, Norton M. E, Wassel Fyr C, Caughey A. B (2006). Unequal placental sharing and birth weight discordance in monochorionic diamniotic twins. Am J Obstet Gynecol.

[14] Burton GJ, Jauniaux E (2018). Pathophysiology of placental-derived fetal growth restriction. Am J Obstet Gynecol.

[15] Brosens I, Puttemans P, Benagiano G (2019). Placental bed research:I. The placental bed:from spiral arteries remodeling to the great obstetrical syndromes. Am J Obstet Gynecol.

